# Domoic Acid: A Review of Its Cytogenotoxicity Within the One Health Approach

**DOI:** 10.3390/antiox13111366

**Published:** 2024-11-08

**Authors:** Goran Gajski, Marko Gerić, Ana Baričević, Mirta Smodlaka Tanković

**Affiliations:** 1 Division of Toxicology, Institute for Medical Research and Occupational Health, 10000 Zagreb, Croatia; 2 Center for Marine Research, Ruđer Bošković Institute, 52210 Rovinj, Croatia

**Keywords:** domoic acid, marine biotoxin, *Pseudo-nitzschia*, non-target cells, cytotoxicity, genotoxicity, oxidative stress, environmental safety, public health, One Health

## Abstract

In this review, we toxicologically assessed the naturally occurring toxin domoic acid. We used the One Health approach because the impact of domoic acid is potentiated by climate change and water pollution on one side, and reflected in animal health, food security, human diet, and human health on the other. In a changing environment, algal blooms are more frequent. For domoic acid production, the growth of *Pseudo-nitzschia* diatoms is of particular interest. They produce this toxin, whose capability of accumulation and biomagnification through the food web impacts other organisms in the ecosystem. Domoic acid targets nervous system receptors inducing amnestic shellfish poisoning, among other less severe health-related problems. However, the impact of domoic acid on non-target cells is rather unknown, so we reviewed the currently available literature on cytogenetic effects on human and animal cells. The results of different studies indicate that domoic acid has the potential to induce early molecular events, such as oxidative imbalance and DNA damage, thus posing an additional threat which needs to be thoroughly addressed and monitored in the future.

## 1. Introduction

The One Health concept integrates and balances the optimal health of humans, animals, plants, and the environment [1]. In light of constant climate change and increase in anthropogenic impact on the planet, more frequent algal blooms are reported causing a threat to all components of the One Health approach and local economies [2,3]. One of the negative aspects of harmful algal blooms is the production of marine toxins [4]. Marine toxins are globally spread natural compounds produced by different phytoplankton microorganisms that readily accumulate in shellfish, finfish, and other marine species and can reach human consumers through the food web. There are numerous marine biotoxins present in the environment such as azaspiracid-1, dinophysistoxin-1, pectenotoxins, yessotoxins, cyclic imines, brevetoxin, ciguatoxin, palytoxin, saxitoxin, tetrodotoxin, okadaic acid, and domoic acid [5]. Many of these toxins may pose a serious threat to animals and humans through poisoning after consuming contaminated seafood, skin contact with contaminated water, and inhaling toxic aerosol chemicals [6,7,8,9].

**Figure 1:**
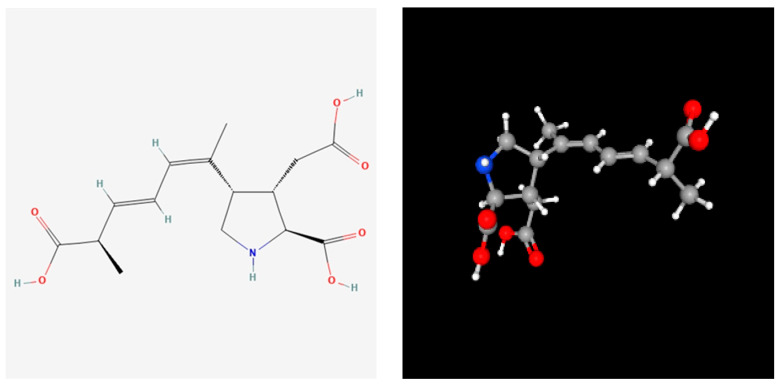
Structure (2D structure (**A**) and 3D conformer (**B**)) of domoic acid. National Center for Biotechnology Information. PubChem Compound Summary for CID 5282253, Domoic acid. https://pubchem.ncbi.nlm.nih.gov/compound/5282253 (accessed on 31 August 2024).

**Figure 2:**
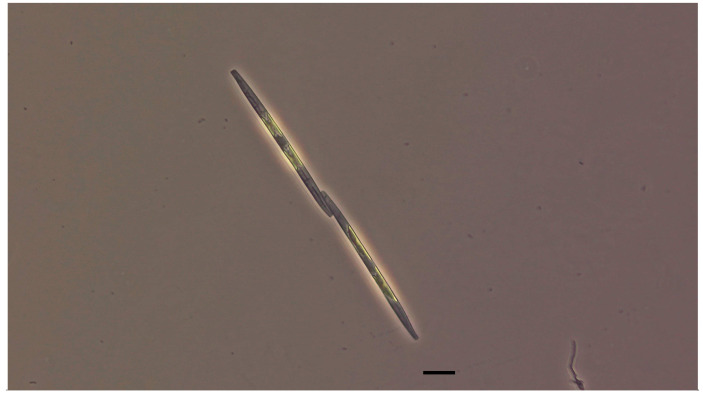
Light micrographs of *Pseudo-nitzschia* species (#CIM1078) from the Adriatic Sea. Culture Collection of the Center for Marine Research, Ruđer Bošković Institute (Rovinj, Croatia; scale 10 μm).

Domoic acid (C_15_H_21_NO_6_, Figure 1) is a naturally occurring biotoxin produced mainly by marine diatoms of the genus *Pseudo-nitzschia* and *Nitzschia* (Figure 2), regarded as cosmopolites widely spread in the worldwide marine phytoplankton community [10,11,12,13], which means that they possess global relevance. Under certain conditions, such as nutrient-rich waters and warmer temperatures, *Pseudo-nitzschia* species can undergo blooms, leading to a significant increase in domoic acid levels in the surrounding waters [11,14,15,16]. These algal blooms can occur seasonally or sporadically and are influenced by factors such as water temperature, nutrient availability, sunlight, and ocean currents. When these blooms occur, shellfish and other filter-feeding organisms can accumulate domoic acid through the consumption of algae or by feeding on other organisms that have already ingested the toxin [17,18,19,20].

Domoic acid can also enter the marine food web through predation, as larger marine animals consume contaminated prey, leading to the biomagnification of the toxin in higher trophic levels [20]. Human activities, such as agricultural runoff and sewage discharge, can exacerbate nutrient input in coastal waters, potentially fueling algal blooms and, in this specific situation, increasing the risk of domoic acid contamination in seafood [21,22,23]. Overall, the occurrence of domoic acid is influenced by a complex interplay of environmental factors, including natural processes and human activities, highlighting the importance of monitoring and managing coastal ecosystems to mitigate the impacts of this potent neurotoxin [24]. To date, the presence of domoic acid has been detected in numerous sea organisms, including dinoflagellates, tunicates, krill, copepods, mollusks such as mussels, oysters and clams, cephalopods, fish, birds, and mammals such as sea lions and whales. Due to domoic acid accumulation, these marine organisms are potential domoic acid vectors to higher trophic levels, but commercially significant species such as finfish, shellfish, and squid are the most significant from the perspective of human health [13,20,25,26,27,28,29].

Domoic acid is primarily a neurotoxic amino acid and glutamate analog that binds to the brain’s glutamate receptors and poses a significant threat to marine life and human health. It is a non-protein amino acid structurally analogous to the excitatory neurotransmitter glutamate, which can induce excitotoxicity in the central nervous system by forming strong bonds with ionotropic glutamate receptors in neural cells. Excessive activation of those receptors leads to the uncontrolled intracellular influx of Ca^2+^ into neurons, resulting in increased oxidative stress, subsequent DNA, lipid, and mitochondrial damage, and ultimately cell death [28,30,31,32,33,34].

Scientific attention first shifted to *Pseudo-nitzschia* species after documenting the amnesic shellfish poisoning (ASP) outbreak that happened on Prince Edward Island (Canada) in 1987, which was connected to these diatoms as the toxin source. A case was defined as the occurrence of gastrointestinal symptoms within 24 h or of neurologic symptoms within 48 h of the ingestion of mussels. There were more than 250 reports received with 107 patients that met the case definition. The most common symptoms were vomiting, abdominal cramps, diarrhea, headache, and loss of short-term memory. Nineteen patients were hospitalized and three patients died [11,35,36]. However, the most famous popular adaptation of ASP remains in Hitchcock’s movie ‘The Birds’ [37,38], where the peculiar behavior of seabirds was related to domoic acid poisoning.

**Figure 3:**
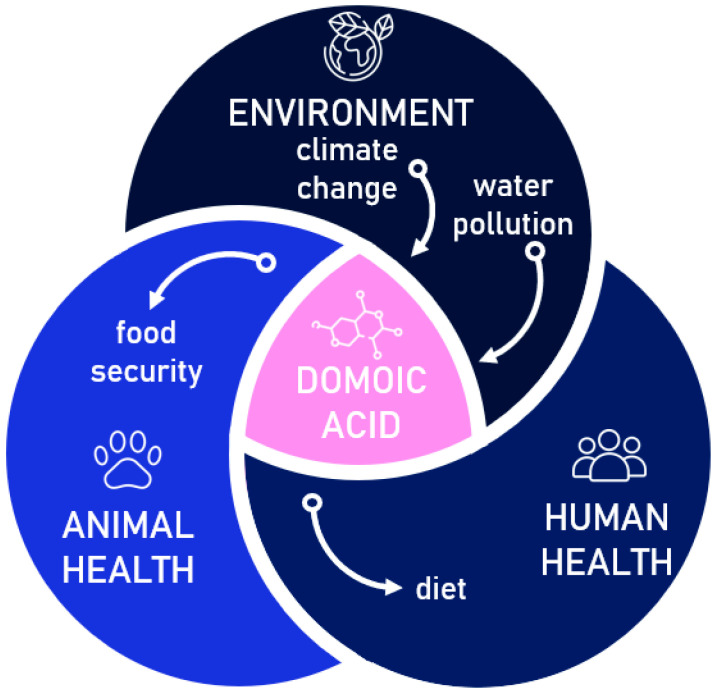
The rationale of evaluating the domoic acid case using the One Health approach, where climate change and increased pollution are major drivers of algal blooms and potential of domoic acid production. The presence of particulate and dissolved domoic acid leads to food contamination, thus impacting animal health, food quality, costs, and human health.

Domoic acid poisoning incidents have been recorded worldwide, with notable outbreaks along the coasts of North America, Europe, and Asia. These incidents often occur due to the consumption of contaminated organisms by humans or animals higher up the food chain [34,39,40,41,42,43]. Within the One Health approach (Figure 3), research efforts should be directed toward better understanding the environmental factors contributing to the proliferation of domoic acid-producing algae and developing effective strategies for more efficient environmental monitoring. These monitoring programs and food contamination regulations would serve to mitigate domoic acid contamination in seafood and help safeguard public health.

## 2. Environmental and Animal Impacts of Domoic Acid

The protection of global health is one of the cornerstones of the One Health approach, yet the anthropocentric point of research has prioritized human health leading to more available data. Therefore, we merged the impacts of domoic acid on environmental and animal levels into one chapter.

A safe environment is the key premise for sustaining an organism’s health. However, there are serious concerns and predictions that climate change, along with global warming and anthropogenic pressures on marine coastal ecosystems, will have dramatic impacts on freshwater and marine environments [44]. The effects mentioned above joined with nutrient input, can cause harmful algal blooms to happen more frequently, in more waterbodies, and with greater intensity [17,45,46]. Climate change can influence the occurrence and distribution of domoic acid in marine environments in several ways. Rising temperatures can promote the growth and proliferation of domoic acid-producing algae, such as *Pseudo-nitzschia* species, because such warmer waters can extend the duration of algal blooms and create more favorable conditions for toxin production, potentially increasing the frequency and intensity of domoic acid contamination events. Alterations in ocean currents and circulation patterns, driven by climate change, can affect the transport and distribution of nutrients and algae in coastal waters. Such changes can in turn influence the dynamics of algal blooms and contribute to the spatial spread of domoic acid contamination. Ocean acidification is another factor contributing to the production of domoic acid. Increasing levels of carbon dioxide (CO_2_) in the atmosphere are leading to ocean acidification, which can impact the physiology and growth of marine algae, including domoic acid-producing species. While the effects of ocean acidification on these algae are complex and still not fully understood, some studies suggest that certain species may benefit from elevated CO_2_ levels, potentially exacerbating domoic acid contamination in the future. Moreover, climate change is expected to increase the frequency and intensity of extreme weather conditions including storms, hurricanes, and heavy rainfalls. All these events can disrupt coastal ecosystems by promoting nutrient runoff from land and creating favorable conditions for algal bloom formation. Consequently, more frequent and severe weather conditions could contribute to increased domoic acid contamination in affected areas [17,30,45,47,48,49,50,51,52,53,54,55,56,57].

On a global scale, algal blooms differ from one region to another with Europe and North America experiencing the largest blooms, whereas Africa and South America tend to have the most frequent ones. The biggest observed increases in frequency have been noticed in some of the major coastal current systems, including the Oyashio Current in the western North Pacific, the Alaska Current, the Malvinas Current off the coast of Patagonia, the Canary Current, the Benguela Current around the coast of southern Africa, and the Gulf Stream. On the contrary, in some places, weakening has been observed over time, including the California Current, parts of the north-eastern North Atlantic, and the Okhotsk Sea in the North Pacific [58,59].

On the contrary, closed marine systems can be more susceptible to harmful algal blooms due to limited water exchange, sheltered environments, nutrient concentrations, warmer water temperatures, or altered salinity compared to open ocean environments. Increased algal bloom frequency and intensity and domoic acid-producing species diversity have been observed in both the Mediterranean and the Adriatic seas, as they are more closed marine systems [11,60,61,62]. Using the Adriatic Sea as an example, where *Pseudo-nitzschia* is found as a dominant and persistent part of the phytoplankton [63,64,65,66,67,68], the presence of shellfish poisoning on the Adriatic coast was first spotted in the year 1989 and was associated with a so-called diarrheic shellfish poisoning. The presence of domoic acid in shellfish was first confirmed in the year 2000. Following that, domoic acid was determined in several shellfish species, though not so often and not reaching regulatory limit concentrations [25,63,69,70,71,72,73,74]. Until now, only four species (*Pseudo-nitzschia delicatissima*, *Pseudo-nitzschia calliantha*, *Pseudo-nitzschia multistriata*, and *Pseudo-nitzschia galaxiae*) from the Adriatic Sea were reported to produce domoic acid in a laboratory culture [25,73,75,76]. *Pseudo-nitzschia* toxicity appears to be strain-dependent, often with geographic partitioning [76]. Nevertheless, due to the constraints of light microscopy and the absence of ongoing molecular genetic monitoring of phytoplankton, the precise species composition and succession remain elusive. As the knowledge about the toxicology and risk posed by individual species continues to expand, the imperative for observation programs tailored to specific species becomes increasingly apparent.

Despite several naturally occurring degradation mechanisms, during intense algal blooms, domoic acid is found in dissolved and particulate form [77,78,79,80,81,82]. When domoic acid enters marine ecosystems, its leads to several significant environmental effects. Since domoic acid is produced by specific species of diatoms, such as *Pseudo-nitzschia*, which can form harmful algal blooms under specific environmental conditions, such blooms can lead to the production and release of large quantities of domoic acid into the water [29,44,45,46,47,48,83]. Domoic acid acts as a stressor for protist communities that are considered important regulators of community structure, microbial activity, and evolution. It triggers cascades of effects in networks and eventually leads to shifts in marine microorganism ecology [83]. Furthermore, it may impact marine life because it can accumulate in various marine organisms such as shellfish, fish, seabirds, and marine mammals, all of which are quite susceptible to domoic acid poisoning. When these organisms ingest contaminated prey or filter-feed on algae containing the toxin, they can experience neurological symptoms, disorientation, seizures, and even mortality, which in turn can severely disrupt marine food webs and impact ecosystem dynamics. In cases of extreme algal blooms, mass animal population strandings and losses were indeed reported, as well as the great contamination of the food web, highlighting additional ecological impacts of the toxin [28,29,32,49,50,51,52,54,55,56,79,84]. In order to protect public health, fishery management authorities may implement closures or restrictions on shellfish harvesting and fishing in areas associated with the synthesis of marine biotoxins and their bioaccumulation. Such closures can have economic repercussions for commercial fisheries and local communities depending on seafood resources [29,85,86,87,88,89].

Overall, harmful algal blooms can disrupt the balance of marine ecosystems, affecting the abundance and distribution of species, nutrient cycling, and ecosystem functioning. Persistent or recurring blooms of domoic acid-producing algae can contribute to long-term environmental degradation and reduce biodiversity in the affected areas. Though related to the environment and animals, one should also have in mind the socio-economic impact of algal blooms, especially on tourism [90,91,92]. Along these lines, there have been several efforts to monitor and mitigate the environmental impacts of domoic acid. These include monitoring water quality, conducting surveillance of algal blooms, implementing early warning systems for harmful algal blooms, and developing strategies to reduce nutrient input and mitigate the factors contributing to bloom formation. By better understanding the environmental effects of domoic acid, researchers and policymakers can work towards the sustainable management of coastal ecosystems and the protection of marine biodiversity.

Altogether, the complex interplay between environmental factors that influence the occurrence and distribution of harmful algal blooms and their associated toxins (such as domoic acid) is potentiated by the changing climate. Understanding these relationships is essential for developing effective strategies to mitigate the occurrence, contamination, and fate of domoic acid in order to minimize its effects on marine ecosystems and animal health.

## 3. Human Health Effects of Domoic Acid

In previous chapters, while explaining the impacts of domoic acid on the environment and animal health, we slightly grazed the human sphere of One Health where the obstruction of certain human activities has led to socio-economic consequences; however, we will now focus on the impacts of domoic acid on human health.

Domoic acid can have severe health effects on humans when ingested through contaminated seafood. The health effects of domoic acid poisoning are known as ASP and can vary depending on the level of exposure and individual susceptibility. In humans and non-human primates, ~2 mg/kg of domoic acid taken orally can induce gastrointestinal disorder symptoms, while a slightly higher concentration can cause different neurological effects. Similar neurotoxic effects have been described for other species ranging from zebrafish to sea lions, as previously reported [32,93,94,95,96].

Some of the key health effects induced by domoic acid include gastrointestinal and neurological symptoms, short-term memory loss, cardiovascular effects, and respiratory problems [27,39,97]. In mild cases of domoic acid poisoning, individuals may experience symptoms such as nausea, vomiting, diarrhea, and abdominal cramps within a few hours of consuming contaminated seafood [6,39]. Since domoic acid primarily affects the central nervous system, intoxication leads to a range of neurological symptoms such as headaches, dizziness, confusion, disorientation, and seizures. In severe cases, individuals may experience hallucinations, tremors, and even coma [39,97,98,99]. The toxin primarily affects the brain, particularly the hippocampus, which is responsible for memory and spatial navigation [10,30,32,33]. One of the hallmark symptoms of ASP is short-term memory loss, which can manifest as a having difficulty recalling recent events, confusion, and disorientation. This symptom can be particularly pronounced and long-lasting, sometimes persisting for weeks or even months after exposure to the toxin [39,100]. In rare cases, domoic acid poisoning may also lead to cardiovascular symptoms such as irregular heartbeat, low blood pressure, and cardiac arrhythmias [6,39,101,102]. Some individuals may experience respiratory distress or difficulty breathing as a result of domoic acid poisoning, although these symptoms are less common compared to the neurological and gastrointestinal effects discussed above [6].

Several animal studies were conducted to clarify the toxicokinetics and toxicodynamics of domoic acid. The oral absorption of domoic acid is approximately 5–10% in adult animals with a low volume of distribution (0.25 L/kg), suggesting that the toxin stays primarily in the blood [103,104]. Domoic acid is mainly excluded from the central nervous system if the blood–brain barrier is intact, but in the case of an immature or defective blood–brain barrier, there is an increased risk of neurologic effects. Domoic acid can also cross the placental barrier in rats and thus enter prenatal brain tissue [103,105,106]. Additionally, also in a rat model, the excretion of domoic acid into the mother’s milk resulted in significant exposure to neonates [103,107].

Domoic acid toxicosis has been linked to cardiovascular, gastrointestinal, and neurologic dysfunction in numerous species. The no-observed-adverse-effect level (NOAEL) in humans has been set at 0.2–0.3 mg/kg, while the lowest-observed-adverse-effect level (LOAEL) is set at about 0.9 mg/kg. Caution should be applied especially for infants, children, pregnant women, and the elderly, all of whom may be more prone to domoic acid toxicosis. In line with the current epidemiological data based on natural outbreaks in humans, several countries including Australia, Canada, the United States of America, and European Union Member States have established maximal allowable levels of domoic acid at 20 mg/kg (20 ppm) of shellfish tissue for human consumption in order to minimize the risk of acute domoic acid exposure and ASP. Nevertheless, lower levels have been suggested by the EFSA (4.5 mg/kg) to accommodate sensitive groups of consumers. However, maximum allowable domoic acid levels for fetuses, infants, and children have still not been established [32,73,103,108,109,110].

Based on the data collected so far, it is important to note that the severity of symptoms can vary widely, and certain individuals, such as the elderly, young children, and individuals with pre-existing medical conditions, may be more vulnerable to the effects of domoic acid poisoning. Prompt medical attention is essential for individuals experiencing symptoms of ASP. Since there is no specific antidote for domoic acid toxicity, medical intervention focuses on addressing symptoms and providing supportive therapy to prevent complications until the toxin is cleared from the body [6,111,112]. The prevention of domoic acid poisoning relies on monitoring and testing seafood to detect contamination, as well as public education and awareness to avoid consuming contaminated shellfish during algal bloom events. Additionally, efforts to mitigate nutrient input and reduce the frequency and intensity of harmful algal blooms can help minimize the risk of domoic acid exposure in affected coastal areas.

## 4. Cytogenotoxic Activity of Domoic Acid

Domoic acid is an excitatory amino acid. Although the neurotoxic effects of domoic acid have been studied well, there is scarce information regarding its potential toxic effects on non-target cells, especially of human origin.

The genotoxic response that domoic acid causes in digestive gland cells has been demonstrated in marine blue mussels (*Mytilus edulis*) in vivo. Primary DNA lesions in the digestive glands of mussels were determined in the acute phase of poisoning within 48 h, and rapidly repaired after 7 days of incubation [113]. Domoic acid also induced significant increases in the frequencies of micronuclei, nuclear abnormalities, and DNA strand breaks in the fish Nile tilapia’s (*Oreochromis niloticus*) peripheral erythrocytes. Samples were evaluated 24, 48, and 72 h post-treatment by the comet and micronucleus assays after treatment with 1, 5, and 10 µg/g b.w. in vivo by intracoelomic injections demonstrating the genotoxic potential of domoic acid [114]. Furthermore, in the same species exposed to three different concentrations (1, 5, and 10 µg/g b.w.) of domoic acid by intraperitoneal injections, changes in the level of lipid peroxidation and activities of antioxidant enzymes such as superoxide dismutase, catalase, glutathione peroxidase, and glutathione reductase were also observed with the effect more pronounced in the liver than in gill tissue [115]. In California sea lions (*Zalophus californianus*) with domoic acid toxicosis, an increased expression of malondialdehyde and 3-nitrotyrosine occurred in neurons of the hippocampal formation indicating oxidative stress [116]. A comparative study on mice (ICR female mice) revealed that in vivo exposure to a single dose of domoic acid (2.5 μg/g b.w.) resulted in a significant increase in monocyte phagocytosis, a significant decrease in both neutrophil and monocyte phagocytosis, and a significant reduction in T-cell mitogen-induced lymphocyte proliferation. On the other hand, in vitro exposure significantly reduced neutrophil and monocyte phagocytosis at 1 μM. B- and T-cell mitogen-induced lymphocyte proliferation were both significantly increased at 1 and 10 μM, and significantly decreased at 100 μM. The observed differences between in vitro and in vivo results indicate that domoic acid may exert its immunotoxic effects both directly and indirectly. The modulation of cytosolic calcium suggests that domoic acid exhibits its effects through ionotropic glutamate subtype surface receptors, at least on monocytes [117]. In addition, the effects of domoic acid on innate and adaptive immune functions were evaluated on peripheral blood leukocytes from California sea lions (*Zalophus californianus*) and southern sea otters (*Enhydra lutris*) in vitro (0.0001–100 µM) and the authors observed that domoic acid did not significantly modulate phagocytosis or respiratory burst in either species. For California sea lions, domoic acid significantly increased ConA-induced T-lymphocyte proliferation (0.0001–10 µM), while there were no effects on lymphocyte proliferation in southern sea otters. The authors concluded that in vitro domoic acid concentrations affecting T-cell proliferation were within or below the range of domoic acid in serum measured in free-ranging California sea lions following natural exposure, suggesting a risk for immunomodulation in free-ranging animals [118].

In cultured cells, domoic acid was able to induce dose-dependent cytotoxicity in human leukemia (K562) cells, human endothelial (EA.hy926) cells, and monkey kidney Vero cells [119]. In addition, domoic acid induced chromosomal abnormalities in human colorectal adenocarcinoma (Caco-2) cells. The formation of micronuclei upon domoic acid treatment was significant at > 30 ng/mL. Micronuclei are small oval bodies with DNA content captured by a nuclear envelope and are spatially separated from the primary nucleus. As such they are linked with chromosome instability, genome rearrangements, and mutagenesis [120,121,122]. In addition, if such a fragmented part of DNA is exposed to the cytoplasm it may subsequently trigger the activation of genes related to the immune system [123]. The majority of micronuclei observed in domoic acid-treated Caco-2 cells proved to be centromere negative indicating that domoic acid induces clastogenic damage but is not aneugenic. The authors concluded that one cannot rule out possible DNA damage of intestinal cells if the studied concentrations are obtained in vivo, since this may happen with concentrations of toxins just below regulatory limits, as in the case of frequent consumption of contaminated shellfish [124]. Moreover, in the same cells (Caco-2), domoic acid decreased cell viability (IC_50_ about 70 ng/mL), induced direct DNA damage (from 15 ng/mL), and caused apoptotic cell death (at 100 ng/mL). The observed apoptosis was bax-dependent and occurred at high concentrations of domoic acid tested. On the contrary, lower concentrations of domoic acid upregulated both bax (pro-apoptotic) and bcl-2 (antiapoptotic) genes at an apparent constant ratio until a sudden decrease in bcl-2 at 100 ng/mL and an increase in bax. The authors concluded that domoic acid decreases cell viability, damages membranes, lysosomes, and mitochondria, and induces apoptosis through upregulation of bax [125]. It was observed that the toxicity induced by domoic acid in Caco-2 cells (IC_50_ about 75 ng/mL) was mediated by oxidative insult leading to morphological changes, DNA damage, and apoptosis. The authors observed an increase in reactive oxygen species generation and nitric oxide production accompanied by significant downregulation in the levels of antioxidant enzymes such as glutathione reductase and catalase [126]. On the contrary, in Chinese hamster lung (V79) fibroblasts, the authors failed to observe an increase in micronuclei frequency or the sister-chromatid exchanges at doses of 27.2 and 54.4 µg/mL with or without activation by freshly isolated rat liver hepatocytes, indicating that domoic acid is non-toxic for V79 cells, within the limits of the test system employed [127]. Domoic acid also did not exhibit any cytotoxic or immunotoxic effect on immature dendritic cells (monocytes and CD34+) at any of the concentrations tested (3.2–320 nmol/L) [128].

Domoic acid also induced cytogenotoxic effects (0.01–10 μg/mL) in human peripheral blood cells evaluated by a battery of bioassays in vitro [129]. The results revealed that domoic acid induced dose- and time-dependent cytotoxicity by affecting cell viability. Domoic acid also significantly affected genomic instability by increasing the frequency of micronuclei and nuclear buds as biomarkers of chromosomal damage [120,121]. Furthermore, a slight induction of primary DNA strand breaks was detected after 24 h of exposure accompanied by a significant increase in the number of abnormal size-tailed nuclei as evaluated by the alkaline comet assay for detection of primary DNA damage [130]. On the contrary, no induction of hOGG1 (human 8-oxoguanine DNA glycosylase) sensitive sites was determined upon in vitro exposure. Additionally, domoic acid induced oxidative stress by increased production of reactive oxygen species accompanied by changes in glutathione, superoxide dismutase, malondialdehyde, and protein carbonyl levels indicating damaging effects towards cells macromolecules such as DNA, lipids, and proteins. Overall, the obtained results showed adverse genotoxic effects of domoic acid in human non-target peripheral blood cells [129].

Additionally, a moderate toxicological response was also observed in the human hepatocellular carcinoma (HepG2) cells treated with domoic acid (0.001–10 μg/mL), where the results showed that domoic acid up to 10 μg/mL did not elicit significant changes in HepG2 cell viability, proliferation, and cell cycle at the applied conditions [131]. Domoic acid also did not generate DNA double-strand breaks as evaluated by the γ-H2AX assay [132], while it did exhibit a significant dose- and time-dependent increase in DNA damage in the form of either DNA single-strand breaks or alkali labile sites as evaluated by the alkaline comet assay. Additionally, increased malondialdehyde levels after domoic acid treatment indicated oxidative damage to lipids. Altogether, the results showed that domoic acid induced only minor adverse genotoxic effects in non-target HepG2 liver cells that most probably occurred resulting from oxidative stress [131].

As previously mentioned, the observed genotoxicity in the above-mentioned studies may be associated with mutagenesis and tumor promotion, which in turn might lead to cancer development causing an additional load on the health system in the ever-increasing aging population [133]. Overall, while the genotoxicity of domoic acid represents a less well-understood effect of its toxicity compared to its neurotoxic effects, it highlights the need for continued investigation into the broader impacts of the toxin on cellular and genetic processes.

An overview of the results on the cytogenotoxic potential of domoic acid on different non-target cells is presented in Table 1.

## 5. Conclusions and Future Directions

In this paper, we aimed to review the importance of domoic acid within the frame of the One Health approach. Usually, the impacts of this marine toxin are evaluated separately, at the level of the environment, animals, or humans; however, as presented here, there is a thin line separating these organizational levels.

From the environmental and animal health perspective, there are severe predictions regarding the profound impacts of climate change, global warming, and human-induced pressures on coastal marine ecosystems in both freshwater and marine environments. Combined with nutrient input, these effects are anticipated to escalate the frequency, extent, and intensity of harmful algal blooms across various water bodies. While algae are considered an important food source for various marine animals, and algal blooms can sometimes be a benefit for ocean ecosystems and fisheries, they can also release toxins into the water bodies and poison the environment in such ways. Therefore, harmful algal blooms are significant threats to marine life, seafood safety, and water quality, thereby endangering different communities of the ecosystem, and the environment [17,24,44,45,46,51,52,54,58,68,134,135,136]. Studies on the topic should focus on two major issues. The first is understanding the complex interaction between abiotic ecological factors, unwanted human activities, and marine algal blooms. The second one is the setup of appropriate biomonitoring systems that will support creating predictive models to be used to predict the occurrence of future domoic acid hotspots. These steps are important for the further preservation of marine ecology and biodiversity, and consecutively human well-being.

An indirect link between domoic acid impact and humans is reflected by obstructing food production due to food contamination and reductions in tourism, all leading to substantial economic costs. Regarding the direct protection of human safety against poisoning from the consumption of contaminated marine organisms, all leading authorities from Europe, the USA, the UK, and Canada set the maximal allowable domoic acid concentration in marine organisms to 20 mg/kg, with the exception of the Dungeness crab in the USA, where the limit is 30 mg/kg. An additional recommendation from UK regulations is not to harvest seafood when more than 1.5 × 10^5^ *Pseudo-nitzschia* cells per liter of seawater is detected. To confirm these limits, the Croatian Agency for Agriculture and Food conducted a risk assessment based on a 6-year surveillance program and concluded that although it is possible to exceed oral acute reference dose (ARfD of 30 µg/kg) from consuming EFSA-recommended portions when the food is contaminated with maximal allowable doses of domoic acid, such a high dose scenario was not observed, and the risk for ASP is negligible [109,110,137,138,139,140].

Based on this overview, additional research is warranted to fully comprehend the mechanisms underlying the toxic effects of domoic acid on non-target cells and possible adverse effects on human health, especially in cases of prolonged exposure to low concentrations. This necessity arises from the recent studies indicating the possible induction of early molecular events such as the induction of oxidative stress, cytotoxicity, and genotoxicity to non-target tissues. A better understanding of these events will enable a more precise risk assessment, which will possibly be reflected in updated legislation. Therefore, a multidisciplinary effort from experts from local and regional to the global level will be needed to tackle the issue of marine toxins in order to promote One Health.

## Figures and Tables

**Table 1:** Summarized results of the cyto/genotoxic potential of domoic acid on non-target cells.

Cell Type	Type of Study	Concentration Range	Method/Assay	Effect	References
Gill cells (*Mytilus edulis*)	In vivo	1–500 ng/g bw	Cholinesterase activity	Positive	Dizer et al. [113]
Digestive gland cells (*Mytilus edulis*)			DNA fragmentation (DNA damage) assay	Positive	
Hemocytes (*Mytilus edulis*)			Cell vitality	Positive	
			Phagocytosis activity	Positive	
Erythrocytes (*Oreochromis niloticus*)	In vivo	1–10 μg/g bw	Comet (DNA damage) assay	Positive	Cavaş and Könen [114]
			Micronucleus (genome instability) assay	Positive	
Liver cells (*Oreochromis niloticus*)	In vivo	1–10 μg/g bw	Lipid peroxidation (LPO) analysis	Positive	Mazmanci and Cavaş [115]
Liver cells (*Oreochromis niloticus*)			Superoxide dismutase (SOD) analysis	Positive	
			Catalase (CAT) analysis	Positive	
			Glutathione peroxidase (GPx) analysis	Positive	
			Glutathione reductase (GR) analysis	Positive	
Gill cells (*Oreochromis niloticus*)			Lipid peroxidation (LPO) analysis	Positive	
			Superoxide dismutase (SOD) analysis	Positive	
			Catalase (CAT) analysis	Positive	
			Glutathione peroxidase (GPx) analysis	Positive	
			Glutathione reductase (GR) analysis	Positive	
Monocytes (ICR female mice)	In vivo	2.5 μg/g bw	Phagocytosis analysis	Positive	Levin et al. [117]
Neutrophils (ICR female mice)					
Lymphocytes (ICR female mice)			Proliferation assay	Positive	
Monocytes (ICR female mice)	In vitro	1–100 μM	Phagocytosis analysis	Positive	
Neutrophils (ICR female mice)					
Lymphocytes (ICR female mice)			Proliferation assay	Positive	
Leukocytes (*Enhydra lutris*)	In vitro	0.0001–100 μM	Phagocytosis and respiratory burst analysis	Negative	Levin et al. [118]
			Proliferation assay	Negative	
Leukocytes (*Zalophus californianus*)			Phagocytosis and respiratory burst analysis	Negative	
			Proliferation assay	Positive	
V79 lung cells (Chinese hamster)	In vitro	27.2 and 54.4 μg/mL	Micronucleus (genome instability) assay	Negative	Rogers and Boyes [127]
			Sister chromatid exchange (SCE) assay	Negative	
Human Caco-2 intestinal cells	In vitro	15–100 ng/mL	Cytotoxicity (MTT) assay	Positive	Carvalho et al. [124]
			Micronucleus (genome instability) assay	Positive	
Human Caco-2 intestinal cells	In vitro	30–300 ng/mL	Cytotoxicity (Trypan Blue) assay	Positive	Carvalho et al. [125]
		15–100 ng/mL	Comet (DNA damage) assay	Positive	
			Apoptotic (AO/EtBr) assay	Positive	
Human Caco-2 intestinal cells	In vitro	10–100 ng/mL	Cytotoxicity (MTT) assay	Positive	Ramya et al. [126]
		75 ng/mL	Apoptotic assay	Positive	
			DNA damage (DAPI) assay	Positive	
			Reactive oxygen species (ROS) analysis	Positive	
			Nitric oxide (NO) analysis	Positive	
			Glutathione reductase (GR) analysis	Positive	
			Catalase (CAT) analysis	Positive	
Human leukemia (K562) cells	In vitro	30–120 μM	Cytotoxicity (MTT and Neutral Red) assay	Positive	Ayed et al. [119]
Human endothelial (EA.hy926) cells					
Monkey kidney Vero cells					
Human dendritic (CD34+ and monocytes) cells	In vitro	3.2–320 nmol/L	Cytotoxicity assay	Negative	Hymery et al. [128]
			Dendritic cell maturation	Negative	
			Cytokine (IL-10 and IL-12) secretion	Negative	
			Autologous lymphocyte proliferation	Negative	
Human peripheral blood cells	In vitro	0.01–10 μg/mL	Cytotoxicity (AO/EtBr) assay and proliferation kinetics	Positive	Gajski et al. [129]
		0.01–1 μg/mL	Comet (DNA damage) assay	Positive	
			hOGG1-modified comet assay	Negative	
			Micronucleus (genome instability) assay	Positive	
			Reactive oxygen species (ROS) analysis	Positive	
			Glutathione (GSH) analysis	Positive	
			Superoxide dismutase (SOD) analysis	Positive	
			Lipid peroxidation (LPO) analysis	Positive	
			Protein carbonyl (PC) analysis	Positive	
Human hepatocellular carcinoma (HepG2) cells	In vitro	0.001–10 μg/mL	Cytotoxicity (MTT) assay	Positive	Madunić et al. [131]
		0.01–1 μg/mL	Proliferation (Ki67) assay	Negative	
			Cell cycle analysis	Negative	
			γ-H2AX (DNA damage) assay	Negative	
			Comet (DNA damage) assay	Positive	
			Lipid peroxidation (LPO) analysis	Positive	

## References

[1] Pitt S.J., Gunn A. (2024). The One Health Concept. Br. J. Biomed. Sci..

[2] Roberts V.A., Vigar M., Backer L., Veytsel G.E., Hilborn E.D., Hamelin E.I., Vanden Esschert K.L., Lively J.Y., Cope J.R., Hlavsa M.C. (2020). Surveillance for Harmful Algal Bloom Events and Associated Human and Animal Illnesses—One Health Harmful Algal Bloom System, United States, 2016–2018. MMWR. Morb. Mortal. Wkly. Rep..

[3] Diogene J. (2017). Marine Toxin Analysis for the Benefit of ‘One Health’ and for the Advancement of Science. Comprehensive Analytical Chemistry.

[4] Turner A.D., Lewis A.M., Bradley K., Maskrey B.H. (2021). Marine Invertebrate Interactions with Harmful Algal Blooms—Implications for One Health. J. Invertebr. Pathol..

[5] Bian Y., Feng X., Zhang Y., Du C., Wen Y. (2024). Marine Toxins in Environment: Recent Updates on Depuration Techniques. Ecotoxicol. Environ. Saf..

[6] Vilariño N., Louzao M., Abal P., Cagide E., Carrera C., Vieytes M., Botana L. (2018). Human Poisoning from Marine Toxins: Unknowns for Optimal Consumer Protection. Toxins.

[7] Gerssen A., Pol-Hofstad I.E., Poelman M., Mulder P.P.J., Van den Top H.J., De Boer J. (2010). Marine Toxins: Chemistry, Toxicity, Occurrence and Detection, with Special Reference to the Dutch Situation. Toxins.

[8] James K.J., Carey B., O’Halloran J., van Pelt F.N.A.M., Škrabáková Z. (2010). Shellfish Toxicity: Human Health Implications of Marine Algal Toxins. Epidemiol. Infect..

[9] Morabito S., Silvestro S., Faggio C. (2018). How the Marine Biotoxins Affect Human Health. Nat. Prod. Res..

[10] Mos L. (2001). Domoic Acid: A Fascinating Marine Toxin. Environ. Toxicol. Pharmacol..

[11] Bates S.S., Hubbard K.A., Lundholm N., Montresor M., Leaw C.P. (2018). Pseudo-Nitzschia, Nitzschia, and Domoic Acid: New Research since 2011. Harmful Algae.

[12] Hasle G.R. (2002). Are Most of the Domoic Acid-Producing Species of the Diatom Genus *Pseudo-nitzschia* Cosmopolites?. Harmful Algae.

[13] Trainer V.L., Bates S.S., Lundholm N., Thessen A.E., Cochlan W.P., Adams N.G., Trick C.G. (2012). Pseudo-Nitzschia Physiological Ecology, Phylogeny, Toxicity, Monitoring and Impacts on Ecosystem Health. Harmful Algae.

[14] Lundholm N., Clarke A., Ellegaard M. (2010). A 100-Year Record of Changing Pseudo-Nitzschia Species in a Sill-Fjord in Denmark Related to Nitrogen Loading and Temperature. Harmful Algae.

[15] Clark S., Hubbard K.A., Anderson D.M., McGillicuddy D.J., Ralston D.K., Townsend D.W. (2019). Pseudo-Nitzschia Bloom Dynamics in the Gulf of Maine: 2012–2016. Harmful Algae.

[16] Hallegraeff G.M. (2010). Ocean Climate Change, Phytoplankton Community Responses, and Harmful Algal Blooms: A Formidable Predictive Challenge. J. Phycol..

[17] WHOI (2022). Harmful Algal Blooms Understanding the Threat and the Actions Being Taken to Address It.

[18] Smodlaka Tanković M., Baričević A., Gerić M., Domijan A.-M., Pfannkuchen D.M., Kužat N., Ujević I., Kuralić M., Rožman M., Matković K. (2022). Characterisation and Toxicological Activity of Three Different Pseudo-Nitzschia Species from the Northern Adriatic Sea (Croatia). Environ. Res..

[19] WHOI (2024). Harrness a National Environmental Science Strategy.

[20] Lefebvre K.A., Bargu S., Kieckhefer T., Silver M.W. (2002). From Sanddabs to Blue Whales: The Pervasiveness of Domoic Acid. Toxicon.

[21] Glibert P.M. (2020). Harmful Algae at the Complex Nexus of Eutrophication and Climate Change. Harmful Algae.

[22] Tsikoti C., Genitsaris S. (2021). Review of Harmful Algal Blooms in the Coastal Mediterranean Sea, with a Focus on Greek Waters. Diversity.

[23] Anderson D., Burkholder J.M., Cochlan W.P., Gobler C.J., Heil C.A., Kudela R.M., Parsons M.L., Rensel J.E.J., Townsend D.W., Trainer V.L. (2008). Harmful Algal Blooms and Eutrophication: Examining Linkages from Selected Coastal Regions of the United States. Harmful Algae.

[24] Anderson D. (2012). HABs in a Changing World: A Perspective on Harmful Algal Blooms, Their Impacts, and Research and Management in a Dynamic Era of Climactic and Environmental Change HHS Public Access. Harmful Algae.

[25] Arapov J., Skejić S., Bužančić M., Bakrač A., Vidjak O., Bojanić N., Ujević I., Gladan Ž.N. (2017). Taxonomical Diversity of *Pseudo-nitzschia* from the Central Adriatic Sea. Phycol. Res..

[26] Ji Y., Yan G., Wang G., Liu J., Tang Z., Yan Y., Qiu J., Zhang L., Pan W., Fu Y. (2022). Prevalence and Distribution of Domoic Acid and Cyclic Imines in Bivalve Mollusks from Beibu Gulf, China. J. Hazard. Mater..

[27] Lefebvre K.A., Robertson A. (2010). Domoic Acid and Human Exposure Risks: A Review. Toxicon.

[28] Bejarano A.C., VanDola F.M., Gulland F.M., Rowles T.K., Schwacke L.H. (2008). Production and Toxicity of the Marine Biotoxin Domoic Acid and Its Effects on Wildlife: A Review. Hum. Ecol. Risk Assess. Int. J..

[29] Broadwater M.H., Van Dolah F.M., Fire S.E. (2018). Vulnerabilities of Marine Mammals to Harmful Algal Blooms. Harmful Algal Blooms.

[30] Brunson J.K., McKinnie S.M.K., Chekan J.R., McCrow J.P., Miles Z.D., Bertrand E.M., Bielinski V.A., Luhavaya H., Oborník M., Smith G.J. (2018). Biosynthesis of the Neurotoxin Domoic Acid in a Bloom-Forming Diatom. Science.

[31] Ohfune Y., Tomita M. (1982). Total Synthesis of (−)-Domoic Acid. A Revision of the Original Structure. J. Am. Chem. Soc..

[32] Costa L.G., Giordano G., Faustman E.M. (2010). Domoic Acid as a Developmental Neurotoxin. Neurotoxicology.

[33] Radad K., Moldzio R., Al-Shraim M., Al-Emam A., Rausch W.-D. (2018). Long-Term Neurotoxic Effects of Domoic Acid on Primary Dopaminergic Neurons. Toxicol. In Vitro.

[34] Jeffery B., Barlow T., Moizer K., Paul S., Boyle C. (2004). Amnesic Shellfish Poison. Food Chem. Toxicol..

[35] Perl T.M., Bédard L., Kosatsky T., Hockin J.C., Todd E.C.D., Remis R.S. (1990). An Outbreak of Toxic Encephalopathy Caused by Eating Mussels Contaminated with Domoic Acid. N. Engl. J. Med..

[36] Wright J.L.C., Boyd R.K., de Freitas A.S.W., Falk M., Foxall R.A., Jamieson W.D., Laycock M.V., McCulloch A.W., McInnes A.G., Odense P. (1989). Identification of Domoic Acid, a Neuroexcitatory Amino Acid, in Toxic Mussels from Eastern Prince Edward Island. Can. J. Chem..

[37] Bargu S., Silver M.W., Ohman M.D., Benitez-Nelson C.R., Garrison D.L. (2012). Mystery behind Hitchcock’s Birds. Nat. Geosci..

[38] Pohnert G., Poulin R.X., Baumeister T.U.H. (2018). The Making of a Plankton Toxin. Science.

[39] Pulido O.M. (2008). Domoic Acid Toxicologic Pathology: A Review. Mar. Drugs.

[40] Saeed A.F., Awan S.A., Ling S., Wang R., Wang S. (2017). Domoic Acid: Attributes, Exposure Risks, Innovative Detection Techniques and Therapeutics. Algal Res..

[41] Zheng G., Wu H., Che H., Li X., Zhang Z., Peng J., Guo M., Tan Z. (2022). Residue Analysis and Assessment of the Risk of Dietary Exposure to Domoic Acid in Shellfish from the Coastal Areas of China. Toxins.

[42] Ben-Gigirey B., Soliño L., Bravo I., Rodríguez F., Casero M.V.M. (2021). Paralytic and Amnesic Shellfish Toxins Impacts on Seabirds, Analyses and Management. Toxins.

[43] Guzick D.S., Overstreet J.W., Factor-Litvak P., Brazil C.K., Nakajima S.T., Coutifaris C., Carson S.A., Cisneros P., Steinkampf M.P., Hill J.A. (2001). Sperm Morphology, Motility, and Concentration in Fertile and Infertile Men. N. Engl. J. Med..

[44] Liquete C., Piroddi C., Macías D., Druon J.-N., Zulian G. (2016). Ecosystem Services Sustainability in the Mediterranean Sea: Assessment of Status and Trends Using Multiple Modelling Approaches. Sci. Rep..

[45] Griffith A.W., Gobler C.J. (2020). Harmful Algal Blooms: A Climate Change Co-Stressor in Marine and Freshwater Ecosystems. Harmful Algae.

[46] Michalak A.M. (2016). Study Role of Climate Change in Extreme Threats to Water Quality. Nature.

[47] Brandenburg K.M., Velthuis M., Van de Waal D.B. (2019). Meta-analysis Reveals Enhanced Growth of Marine Harmful Algae from Temperate Regions with Warming and Elevated CO_2_ Levels. Glob. Chang. Biol..

[48] Fu F., Tatters A., Hutchins D. (2012). Global Change and the Future of Harmful Algal Blooms in the Ocean. Mar. Ecol. Prog. Ser..

[49] Trainer V.L., Moore S.K., Hallegraeff G., Kudela R.M., Clement A., Mardones J.I., Cochlan W.P. (2020). Pelagic Harmful Algal Blooms and Climate Change: Lessons from Nature’s Experiments with Extremes. Harmful Algae.

[50] Wells M.L., Trainer V.L., Smayda T.J., Karlson B.S.O., Trick C.G., Kudela R.M., Ishikawa A., Bernard S., Wulff A., Anderson D.M. (2015). Harmful Algal Blooms and Climate Change: Learning from the Past and Present to Forecast the Future. Harmful Algae.

[51] Cressey D. (2017). Climate Change Is Making Algal Blooms Worse. Nature.

[52] Gobler C.J. (2020). Climate Change and Harmful Algal Blooms: Insights and Perspective. Harmful Algae.

[53] Xu D., Zheng G., Brennan G., Wang Z., Jiang T., Sun K., Fan X., Bowler C., Zhang X., Zhang Y. (2023). Plastic responses lead to increased neurotoxin production in the diatom Pseudo-nitzschia under ocean warming and acidification. ISME J..

[54] Moore S.K., Trainer V.L., Mantua N.J., Parker M.S., Laws E.A., Backer L.C., Fleming L.E. (2008). Impacts of Climate Variability and Future Climate Change on Harmful Algal Blooms and Human Health. Environ. Health.

[55] Townhill B.L., Tinker J., Jones M., Pitois S., Creach V., Simpson S.D., Dye S., Bear E., Pinnegar J.K. (2018). Harmful Algal Blooms and Climate Change: Exploring Future Distribution Changes. ICES J. Mar. Sci..

[56] Armbrust E.V. (2009). The Life of Diatoms in the World’s Oceans. Nature.

[57] Sun J., Hutchins D.A., Feng Y., Seubert E.L., Caron D.A., Fu F.-X. (2011). Effects of Changing *p* CO _2_ and Phosphate Availability on Domoic Acid Production and Physiology of the Marine Harmful Bloom Diatom *Pseudo-nitzschia multiseries*. Limnol. Oceanogr..

[58] Dai Y., Yang S., Zhao D., Hu C., Xu W., Anderson D.M., Li Y., Song X.-P., Boyce D.G., Gibson L. (2023). Coastal Phytoplankton Blooms Expand and Intensify in the 21st Century. Nature.

[59] Harvey C. Algal Blooms Have Boomed Worldwide|Scientific American. https://www.scientificamerican.com/article/algal-blooms-have-boomed-worldwide/.

[60] Hassoun A.E.R., Ujević I., Mahfouz C., Fakhri M., Roje-Busatto R., Jemaa S., Nazlić N. (2021). Occurrence of Domoic Acid and Cyclic Imines in Marine Biota from Lebanon-Eastern Mediterranean Sea. Sci. Total Environ..

[61] Marić D., Kraus R., Godrijan J., Supić N., Djakovac T., Precali R. (2012). Phytoplankton Response to Climatic and Anthropogenic Influences in the North-Eastern Adriatic during the Last Four Decades. Estuar. Coast. Shelf Sci..

[62] Turk Dermastia T., Cerino F., Stanković D., Francé J., Ramšak A., Žnidarič Tušek M., Beran A., Natali V., Cabrini M., Mozetič P. (2020). Ecological Time Series and Integrative Taxonomy Unveil Seasonality and Diversity of the Toxic Diatom *Pseudo-nitzschia* H. Peragallo in the Northern Adriatic Sea. Harmful Algae.

[63] Ljubešić Z., Bosak S., Viličić D., Borojević K.K., Marić D., Godrijan J., Ujević I., Peharec P., Đakovac T. (2011). Ecology and Taxonomy of Potentially Toxic *Pseudo-nitzschia* Species in Lim Bay (North-Eastern Adriatic Sea). Harmful Algae.

[64] Marić D., Ljubešić Z., Godrijan J., Viličić D., Ujević I., Precali R. (2011). Blooms of the Potentially Toxic Diatom *Pseudo-nitzschia* Calliantha Lundholm, Moestrup & Hasle in Coastal Waters of the Northern Adriatic Sea (Croatia). Estuar. Coast. Shelf Sci..

[65] Orsini L., Sarno D., Procaccini G., Poletti R., Dahlmann J., Montresor M. (2002). Toxic *Pseudo-nitzschia* Multistriata (Bacillariophyceae) from the Gulf of Naples: Morphology, Toxin Analysis and Phylogenetic Relationships with Other *Pseudo-nitzschia* Species. Eur. J. Phycol..

[66] Viličić D., Djakovac T., Burić Z., Bosak S. (2009). Composition and Annual Cycle of Phytoplankton Assemblages in the Northeastern Adriatic Sea. Bot. Mar..

[67] Quiroga I. (2006). *Pseudo-nitzschia* Blooms in the Bay of Banyuls-Sur-Mer, Northwestern Mediterranean Sea. Diatom Res..

[68] Zingone A., Escalera L., Aligizaki K., Fernández-Tejedor M., Ismael A., Montresor M., Mozetič P., Taş S., Totti C. (2021). Toxic Marine Microalgae and Noxious Blooms in the Mediterranean Sea: A Contribution to the Global HAB Status Report. Harmful Algae.

[69] Arapov J., Ujević I., Marić Pfankuchen D., Godrijan J., Bakrač A., Ninčević Gladan Ž., Marasović I. (2016). Domoic Acid in Phytoplankton Net Samples and Shellfish from the Krka River Estuary in the Central Adriatic Sea. Mediterr. Mar. Sci..

[70] Ciminiello P., Dell’Aversano C., Fattorusso E., Forino M., Magno G.S., Tartaglione L., Grillo C., Melchiorre N. (2006). The Genoa 2005 Outbreak. Determination of Putative Palytoxin in Mediterranean Ostreopsis Ovata by a New Liquid Chromatography Tandem Mass Spectrometry Method. Anal. Chem..

[71] Della Loggia R., Cabrini M., Del Negro P., Honsell G., Tubaro A., Smayda T.J., Shimizu Y. (1993). Relationships between *Dinophysis* Spp. in Seawater and DSP Toxins in Mussels in the Northern Adriatic Sea. Toxic Phytoplankton Blooms in the Sea.

[72] Marasović I., Ninčević Ž., Pavela-Vrančič M., Orhanović S. (1998). A Survey of Shellfish Toxicity in the Central Adriatic Sea. J. Mar. Biol. Assoc. UK.

[73] Pistocchi R., Guerrini F., Pezzolesi L., Riccardi M., Vanucci S., Ciminiello P., Dell’Aversano C., Forino M., Fattorusso E., Tartaglione L. (2012). Toxin Levels and Profiles in Microalgae from the North-Western Adriatic Sea—15 Years of Studies on Cultured Species. Mar. Drugs.

[74] Ujević I., Ninčević-Gladan Z., Roje R., Skejić S., Arapov J., Marasović I. (2010). Domoic Acid—A New Toxin in the Croatian Adriatic Shellfish Toxin Profile. Molecules.

[75] Penna A., Casabianca S., Perini F., Bastianini M., Riccardi E., Pigozzi S., Scardi M. (2013). Toxic *Pseudo-nitzschia* Spp. in the Northwestern Adriatic Sea: Characterization of Species Composition by Genetic and Molecular Quantitative Analyses. J. Plankton Res..

[76] Turk Dermastia T., Dall’Ara S., Dolenc J., Mozetič P. (2022). Toxicity of the Diatom Genus *Pseudo-nitzschia* (Bacillariophyceae): Insights from Toxicity Tests and Genetic Screening in the Northern Adriatic Sea. Toxins.

[77] Li Z., Wang J., Yousaf M., Rehman A., Wang F. (2024). Biogenic ROS Mediated Degradation Mechanism of Marine Toxin Domoic Acid. Sci. Total Environ..

[78] Jaramillo M., Joens J.A., O’Shea K.E. (2020). Fundamental Studies of the Singlet Oxygen Reactions with the Potent Marine Toxin Domoic Acid. Environ. Sci. Technol..

[79] Smith J., Connell P., Evans R.H., Gellene A.G., Howard M.D.A., Jones B.H., Kaveggia S., Palmer L., Schnetzer A., Seegers B.N. (2018). A Decade and a Half of *Pseudo-nitzschia* Spp. and Domoic Acid along the Coast of Southern California. Harmful Algae.

[80] Perry R.I., Nemcek N., Hennekes M., Sastri A., Ross A.R.S., Shannon H., Shartau R.B. (2023). Domoic Acid in Canadian Pacific Waters, from 2016 to 2021, and Relationships with Physical and Chemical Conditions. Harmful Algae.

[81] Shartau R.B., Turcotte L.D.M., Bradshaw J.C., Ross A.R.S., Surridge B.D., Nemcek N., Johnson S.C. (2023). Dissolved Algal Toxins along the Southern Coast of British Columbia Canada. Toxins.

[82] Trapp A., Hayashi K., Fiechter J., Kudela R.M. (2023). What Happens in the Shadows—Influence of Seasonal and Non-Seasonal Dynamics on Domoic Acid Monitoring in the Monterey Bay Upwelling Shadow. Harmful Algae.

[83] Li Z., Wang J., Fan J., Yue H., Zhang X. (2023). Marine Toxin Domoic Acid Alters Protistan Community Structure and Assembly Process in Sediments. Mar. Environ. Res..

[84] McKibben S.M., Peterson W., Wood A.M., Trainer V.L., Hunter M., White A.E. (2017). Climatic Regulation of the Neurotoxin Domoic Acid. Proc. Natl. Acad. Sci. USA.

[85] Zabaglo K., Chrapusta E., Bober B., Kaminski A., Adamski M., Bialczyk J. (2016). Environmental Roles and Biological Activity of Domoic Acid: A Review. Algal Res..

[86] Chenouf S., Pérez Agúndez J.A., Raux P. (2023). Analysing the Socioeconomic Impacts of Fishing Closures Due to Toxic Algal Blooms: Application of the Vulnerability Framework to the Case of the Scallop Fishery in the Eastern English Channel. Sustainability.

[87] Rolton A., Rhodes L., Hutson K.S., Biessy L., Bui T., MacKenzie L., Symonds J.E., Smith K.F. (2022). Effects of Harmful Algal Blooms on Fish and Shellfish Species: A Case Study of New Zealand in a Changing Environment. Toxins.

[88] Oh J.-W., Pushparaj S.S.C., Muthu M., Gopal J. (2023). Review of Harmful Algal Blooms (HABs) Causing Marine Fish Kills: Toxicity and Mitigation. Plants.

[89] Moore S.K., Cline M.R., Blair K., Klinger T., Varney A., Norman K. (2019). An Index of Fisheries Closures Due to Harmful Algal Blooms and a Framework for Identifying Vulnerable Fishing Communities on the U.S. West Coast. Mar. Policy.

[90] Alvarez S., Brown C.E., Garcia Diaz M., O’Leary H., Solís D. (2024). Non-Linear Impacts of Harmful Algae Blooms on the Coastal Tourism Economy. J. Environ. Manag..

[91] Sanseverino I., Conduto D., Pozzoli L., Dobricic S., Lettieri T. (2016). Algal bloom and its economic impact. Eur. Comm. Jt. Res. Cent. Inst. Environ. Sustain..

[92] CDC (2023). Harmful Algal Blooms Threaten Our Health, Environment, and Economy.

[93] Lefebvre K.A., Tilton S.C., Bammler T.K., Beyer R.P., Srinouanprachan S., Stapleton P.L., Farin F.M., Gallagher E.P. (2009). Gene Expression Profiles in Zebrafish Brain after Acute Exposure to Domoic Acid at Symptomatic and Asymptomatic Doses. Toxicol. Sci..

[94] Scholin C.A., Gulland F., Doucette G.J., Benson S., Busman M., Chavez F.P., Cordaro J., DeLong R., De Vogelaere A., Harvey J. (2000). Mortality of Sea Lions along the Central California Coast Linked to a Toxic Diatom Bloom. Nature.

[95] Silvagni P.A., Lowenstine L.J., Spraker T., Lipscomb T.P., Gulland F.M.D. (2005). Pathology of Domoic Acid Toxicity in California Sea Lions (*Zalophus californianus*). Vet. Pathol..

[96] Tryphonas L., Truelove J., Todd E., Nera E., Iverson F. (1990). Experimental Oral Toxicity of Domoic Acid in Cynomolgus Monkeys (*Macaca fascicularis*) and Rats. Preliminary Investigations. Food Chem. Toxicol..

[97] Petroff R., Hendrix A., Shum S., Grant K.S., Lefebvre K.A., Burbacher T.M. (2021). Public Health Risks Associated with Chronic, Low-Level Domoic Acid Exposure: A Review of the Evidence. Pharmacol. Ther..

[98] Ramsdell J.S., Gulland F.M. (2014). Domoic Acid Epileptic Disease. Mar. Drugs.

[99] Grant K.S., Burbacher T.M., Faustman E.M., Gratttan L. (2010). Domoic Acid: Neurobehavioral Consequences of Exposure to a Prevalent Marine Biotoxin. Neurotoxicol. Teratol..

[100] Grattan L.M., Kaddis L., Tracy J.K., Morris J.G. (2021). Long Term Memory Outcome of Repetitive, Low-Level Dietary Exposure to Domoic Acid in Native Americans. Int. J. Environ. Res. Public Health.

[101] Teitelbaum J.S., Zatorre R.J., Carpenter S., Gendron D., Evans A.C., Gjedde A., Cashman N.R. (1990). Neurologic Sequelae of Domoic Acid Intoxication Due to the Ingestion of Contaminated Mussels. N. Engl. J. Med..

[102] Todd E.C.D. (1993). Domoic Acid and Amnesic Shellfish Poisoning—A Review. J. Food Prot..

[103] Gwaltney-Brant S.M. (2017). Zootoxins. Reproductive and Developmental Toxicology.

[104] Ramsdell J.S., Zabka T.S. (2008). In Utero Domoic Acid Toxicity: A Fetal Basis to Adult Disease in the California Sea Lion (Zalophus Californianus). Mar. Drugs.

[105] Maucher J.M., Ramsdell J.S. (2007). Maternal–Fetal Transfer of Domoic Acid in Rats at Two Gestational Time Points. Environ. Health Perspect..

[106] Tanemura K., Igarashi K., Matsugami T.-R., Aisaki K., Kitajima S., Kanno J. (2009). Intrauterine Environment-Genome Interaction and Children’s Development (2): Brain Structure Impairment and Behavioral Disturbance Induced in Male Mice Offspring by a Single Intraperitoneal Administration of Domoic Acid (DA) to Their Dams. J. Toxicol. Sci..

[107] Maucher J.M., Ramsdell J.S. (2005). Domoic Acid Transfer to Milk: Evaluation of a Potential Route of Neonatal Exposure. Environ. Health Perspect..

[108] Kumar K.P., Kumar S.P., Nair G.A. (2009). Risk Assessment of the Amnesic Shellfish Poison, Domoic Acid, on Animals and Humans. J. Environ. Biol..

[109] Paredes I., Rietjens I.M.C.M., Vieites J.M., Cabado A.G. (2011). Update of Risk Assessments of Main Marine Biotoxins in the European Union. Toxicon.

[110] Alexander J., Benford D., Boobis A., Ceccatelli S., Cravedi J., Domenico A.D., Doerge D., Dogliotti E., Edler L., Farmer P. (2009). Marine Biotoxins in Shellfish—Domoic Acid Scientific Opinion of the Panel on Contaminants in the Food Chain Adopted on 2 July 2009. EFSA J..

[111] Lee M.J., Henderson S.B., Clermont H., Turna N.S., McIntyre L. (2024). The Health Risks of Marine Biotoxins Associated with High Seafood Consumption: Looking beyond the Single Dose, Single Outcome Paradigm with a View towards Addressing the Needs of Coastal Indigenous Populations in British Columbia. Heliyon.

[112] Schroeder G., Bates S.S., Spallino J. (2015). Amnesic Shellfish Poisoning: Emergency Medical Management. J. Mar. Sci. Res. Dev..

[113] Dizer H., Fischer B., Harabawy A.S., Hennion M.C., Hansen P.D. (2001). Toxicity of Domoic Acid in the Marine Mussel Mytilus Edulis. Aquat. Toxicol..

[114] Cavaş T., Könen S. (2008). In Vivo Genotoxicity Testing of the Amnesic Shellfish Poison (Domoic Acid) in Piscine Erythrocytes Using the Micronucleus Test and the Comet Assay. Aquat. Toxicol..

[115] Mazmanci B., Cavaş T. (2010). Antioxidant Enzyme Activity and Lipid Peroxidation in Liver and Gill Tissues of Nile Tilapia (*Oreochromis niloticus*) Following in Vivo Exposure to Domoic Acid. Toxicon.

[116] Madl J.E., Duncan C.G., Stanhill J.E., Tai P.-Y., Spraker T.R., Gulland F.M. (2014). Oxidative Stress and Redistribution of Glutamine Synthetase in California Sea Lions (*Zalophus californianus*) with Domoic Acid Toxicosis. J. Comp. Pathol..

[117] Levin M., Leibrecht H., Ryan J., Van Dolah F., De Guise S. (2008). Immunomodulatory Effects of Domoic Acid Differ between in Vivo and in Vitro Exposure in Mice. Mar. Drugs.

[118] Levin M., Joshi D., Draghi A., Gulland F.M., Jessup D., De Guise S. (2010). Immunomodulatory Effects upon in Vitro Exposure of California Sea Lion and Southern Sea Otter Peripheral Blood Leukocytes to Domoic Acid. J. Wildl. Dis..

[119] Ayed Y., Kouidhi B., Kassim S., Bacha H. (2018). Proliferative Effect of the Phycotoxin Domoic Acid on Cancer Cell Lines: A Preliminary Evaluation. J. Taibah Univ. Sci..

[120] Gajski G., Gerić M., Oreščanin V., Garaj-Vrhovac V. (2018). Cytokinesis-Block Micronucleus Cytome Assay Parameters in Peripheral Blood Lymphocytes of the General Population: Contribution of Age, Sex, Seasonal Variations and Lifestyle Factors. Ecotoxicol. Environ. Saf..

[121] Gajski G., Kašuba V., Milić M., Gerić M., Matković K., Delić L., Nikolić M., Pavičić M., Rozgaj R., Garaj-Vrhovac V. (2024). Exploring Cytokinesis Block Micronucleus Assay in Croatia: A Journey through the Past, Present, and Future in Biomonitoring of the General Population. Mutat. Res. Genet. Toxicol. Environ. Mutagen..

[122] Kopjar N., Kašuba V., Milić M., Rozgaj R., Želježić D., Gajski G., Mladinić M., Garaj-Vrhovac V. (2010). Normal and Cut-off Values of the Cytokinesis-Block Micronucleus Assay on Peripheral Blood Lymphocytes in the Croatian General Population. Arh. Hig. Rada Toksikol..

[123] Gekara N.O. (2017). DNA Damage-Induced Immune Response: Micronuclei Provide Key Platform. J. Cell Biol..

[124] Carvalho P.-S., Catia R., Moukha S., Matias W.G., Creppy E.E. (2006). Comparative Study of Domoic Acid and Okadaic Acid Induced—Chromosomal Abnormalities in the CACO-2 Cell Line. Int. J. Environ. Res. Public Health.

[125] Carvalho Pinto-Silva C.R., Moukha S., Matias W.G., Creppy E.E. (2008). Domoic Acid Induces Direct DNA Damage and Apoptosis in Caco-2 Cells: Recent Advances. Environ. Toxicol..

[126] Ramya E.M., Kumar G.P., Anand T., Anilakumar K.R. (2017). Modulatory Effects of Terminalia Arjuna against Domoic Acid Induced Toxicity in Caco-2 Cell Line. Cytotechnology.

[127] Rogers C.G., Boyes B.G. (1989). Evaluation of the Genotoxicity of Domoic Acid in a Hepatocyte-Mediated Assay with V79 Chinese Hamster Lung Cells. Mutat. Res..

[128] Hymery N., Sibiril Y., Parent-Massin D. (2006). Improvement of Human Dendritic Cell Culture for Immunotoxicological Investigations. Cell Biol. Toxicol..

[129] Gajski G., Gerić M., Domijan A.-M., Golubović I., Žegura B. (2020). Marine Toxin Domoic Acid Induces in Vitro Genomic Alterations in Human Peripheral Blood Cells. Toxicon.

[130] Collins A., Møller P., Gajski G., Vodenková S., Abdulwahed A., Anderson D., Bankoglu E.E., Bonassi S., Boutet-Robinet E., Brunborg G. (2023). Measuring DNA Modifications with the Comet Assay: A Compendium of Protocols. Nat. Protoc..

[131] Madunić J., Hercog K., Gerić M., Domijan A.-M., Žegura B., Gajski G. (2022). Marine Toxin Domoic Acid Induces Moderate Toxicological Response in Non-Target HepG2 Cells. Toxicology.

[132] Gerić M., Gajski G., Garaj-Vrhovac V. (2014). γ-H2AX as a Biomarker for DNA Double-Strand Breaks in Ecotoxicology. Ecotoxicol. Environ. Saf..

[133] Viegas S., Ladeira C., Costa-Veiga A., Perelman J., Gajski G. (2017). Forgotten Public Health Impacts of Cancer—An Overview. Arh. Hig. Rada Toksikol..

[134] Tewari K. (2022). A Review of Climate Change Impact Studies on Harmful Algal Blooms. Phycology.

[135] Hallegraeff G.M., Anderson D.M., Belin C., Bottein M.-Y.D., Bresnan E., Chinain M., Enevoldsen H., Iwataki M., Karlson B., McKenzie C.H. (2021). Perceived Global Increase in Algal Blooms Is Attributable to Intensified Monitoring and Emerging Bloom Impacts. Commun. Earth Environ..

[136] Dale B., Edwards M., Reid P.C. (2006). Climate Change and Harmful Algal Blooms. Ecology of Harmful Algae.

[137] Hengl B., Petrić J., Gross Bošković A., Ujević I., Listeš E., Bogdanović T., Džafić N., Kvrgić K. (2024). Znanstveno Mišljenje o Morskim Biotoksinima u Školjkašima u Republici Hrvatskoj.

[138] Toyofuku H. (2006). Joint FAO/WHO/IOC Activities to Provide Scientific Advice on Marine Biotoxins (Research Report). Mar. Pollut. Bull..

[139] FDA (2021). Appendix 5: FDA and EPA Safety Levels in Regulations and Guidance.

[140] FSA (2023). Biotoxin and Phytoplankton Monitoring.

